# Primary Transgenic Bovine Cells and Their Rejuvenated Cloned Equivalents Show Transgene-Specific Epigenetic Differences

**DOI:** 10.1371/journal.pone.0035619

**Published:** 2012-04-20

**Authors:** Lucia Alonso-González, Christine Couldrey, Marcus W. Meinhardt, Sally A. Cole, David N. Wells, Götz Laible

**Affiliations:** AgResearch Ruakura Research Centre, Hamilton, New Zealand; Bellvitge Biomedical Research Institute (IDIBELL), Spain

## Abstract

Cell-mediated transgenesis, based on somatic cell nuclear transfer (SCNT), provides the opportunity to shape the genetic make-up of cattle. Bovine primary fetal fibroblasts, commonly used cells for SCNT, have a limited lifespan, and complex genetic modifications that require sequential transfections can be challenging time and cost-wise. To overcome these limitations, SCNT is frequently used to rejuvenate the cell lines and restore exhausted growth potential. We have designed a construct to be used in a 2-step cassette exchange experiment. Our transgene contains a puromycin resistance marker gene and an enhanced green fluorescence protein (EGFP) expression cassette, both driven by a strong mammalian promoter, and flanked by loxP sites and sequences from the bovine β-casein locus. Several transgenic cell lines were generated by random insertion into primary bovine cell lines. Two of these original cell lines were rederived by SCNT and new primary cells, with the same genetic makeup as the original donors, were established. While the original cell lines were puromycin-resistant and had a characteristic EGFP expression profile, all rejuvenated cell lines were sensitive to puromycin, and displayed varied EGFP expression, indicative of various degrees of silencing. When the methylation states of individual CpG sites within the transgene were analyzed, a striking increase in transgene-specific methylation was observed in all rederived cell lines. The results indicate that original transgenic donor cells and their rejuvenated derivatives may not be equivalent and differ in the functionality of their transgene sequences.

## Introduction

The technique of SCNT, whereby a clonal embryo is generated after the fusion of a somatic cell with an enucleated oocyte, has been successfully used to generate cloned and transgenic animals in numerous species, and is the preferred tool to achieve modification of the genetic make-up of cattle. However, the generation of transgenic animals other than mice, especially those that require targeted insertion, still remains a lengthy and inefficient process [1] due to the unavailability of embryonic stem cells in mammalian species other than mice and rats. Primary fetal fibroblasts remain the preferred cell type to be used for the generation of transgenic cattle using SCNT. However, these primary cell lines lack the ability to replicate indefinitely and struggle to recover after sequential genetic modifications. To overcome these problems, cell rederivation from SCNT fetuses has often been used as a method to restore proliferative potential when sequential genome modifications are required. However, although the genetic makeup of cells rederived by SCNT is identical to that of their original donor cells, the two cell lines are not completely equivalent. Due to the reprogramming process the cells are subjected to during and after nuclear transfer, epigenetic differences can emerge that affect gene expression [2], [3], [4], including that of the transgene. Some studies involving several rounds of cell rejuvenation using SCNT have found evidence of transgene-specific silencing after reprogramming [5]. Epigenetic changes have been proposed as the cause of this variation in gene expression, and DNA methylation has frequently been suggested as the mechanism mainly responsible for transgene silencing. However, due to the lack of reliable methods to determine specific CpG methylation the role of DNA methylation in causing impaired expression of transgenes is not well understood. Recently though, affordable and relatively uncomplicated methods have been devised that allow for the accurate and reproducible determination of the methylation status at individual CpGs of known sequences. The most accurate of these methods is known as quantitative high-throughput mass spectrometry (QHTMS) [6], which allows accurate and precise quantification of methylation levels at individual CpG sites along a known sequence [6], [7].

In the present study, QHTMS was used to investigate DNA methylation patterns in a transgene containing sequences from various origins in primary bovine fibroblasts rederived by SCNT. We find that CpG methylation levels increase markedly in transgene sequences in all rederived cell lines and transgene expression is altered compared with the original donor cell lines. From these results, it can be implied that epigenetic processes that occur during embryonic development can influence functionality of transgene sequences which can exclude the reliable extrapolation of functional attributes from transgenic donor cell lines to SCNT derived rejuvenated cells or transgenic animals.

## Results

**Figure 1:**
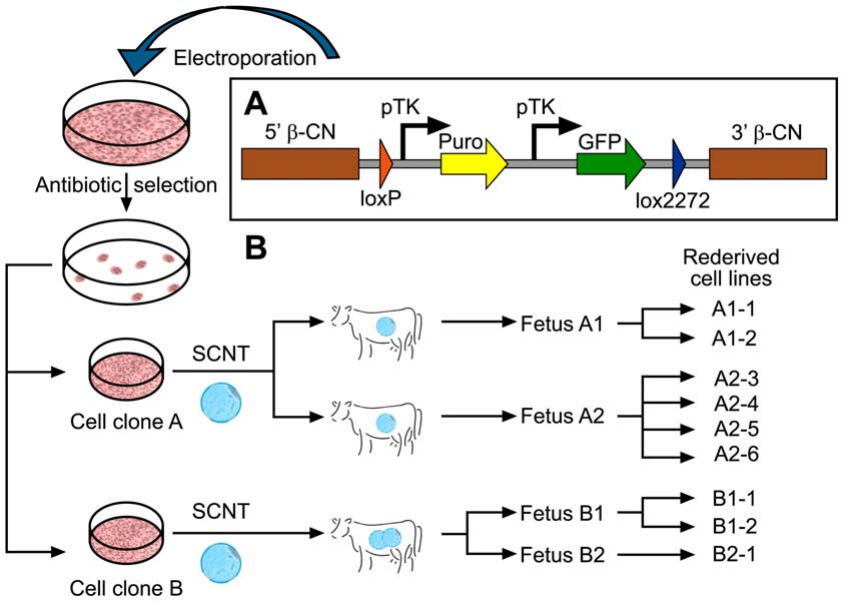
Transgene construct and cell line isolation and rederivation scheme. (a) The transgene construct is comprised of a puromycin-resistance gene (Puro) and an EGFP gene (EGFP), each driven by an individual TK promoter (pTK) and flanked firstly by a pair of incompatible lox sites (loxP, lox2272) and secondly homologous sequence arms of the bovine beta-casein promoter (5′ β-CN) and 3′ untranslated region (3′ β-CN). (b) Primary bovine cells were transfected and cell clones isolated following antibiotic selection. Two cell clones (A and B) were chosen as source for donor cells to generate embryos using SCNT. Following transfer into recipient cows for further in vivo development, two resulting fetuses generated from cell line A (fetus A1, A2) and cell line B (fetus B1 and B2) were used to isolate a series of rejuvenated primary cell lines with the same genetics as the original cell lines A (A1-1, A1-2, A2-3, A2-4, A2-5, A2-6) and B (B1-1, B1-2, B2-1).

As previously published [8], several transgenic cell lines were generated by random insertion of a cassette exchange construct (Fig. 1a) into primary bovine fibroblasts. All isolated transgenic cell lines were puromycin-resistant, showed varying, albeit characteristic EGFP expression profiles and were assessed by quantitative real-time PCR to determine the number of integrated transgene copies (data not shown).

Two of these cell lines, A and B, each originating from an independent insertion event and hence containing unique insertion sites, were selected as acceptor cell lines based on the integration of only one or two transgene copies. Subsequently, both were used as donor cells for SCNT in an attempt to rederive cell lines with rejuvenated proliferative capacity that were genetically and functionally equivalent to the original cell lines A and B. A total of six cell lines were derived from two SCNT fetuses (A1 and A2) generated using cell line A and three cell lines from two SCNT fetuses B1 and B2 produced using cell line B (Fig. 1b). The cell lines represent cell populations isolated from the embryo proper (A1-1, A1-2, A2-3, B1-1, B2-1) as well as extra embryonic cells and tissues (A2-4, A2-5, A2-6 and B1-2).

### Transgene expression characteristics of original and rederived cell lines

To evaluate the similarity of original and rederived cell lines, their transgene expression characteristics were initially compared. Under puromycin selection, original cell lines A and B proliferated and remained healthy for the entire selection period, indicative of a resistant phenotype. In contrast, all cells from rederived cell lines cultured under puromycin selection were dead and had lifted off the surface of the culture dish 72 hours after the addition of puromycin.

**Figure 2:**
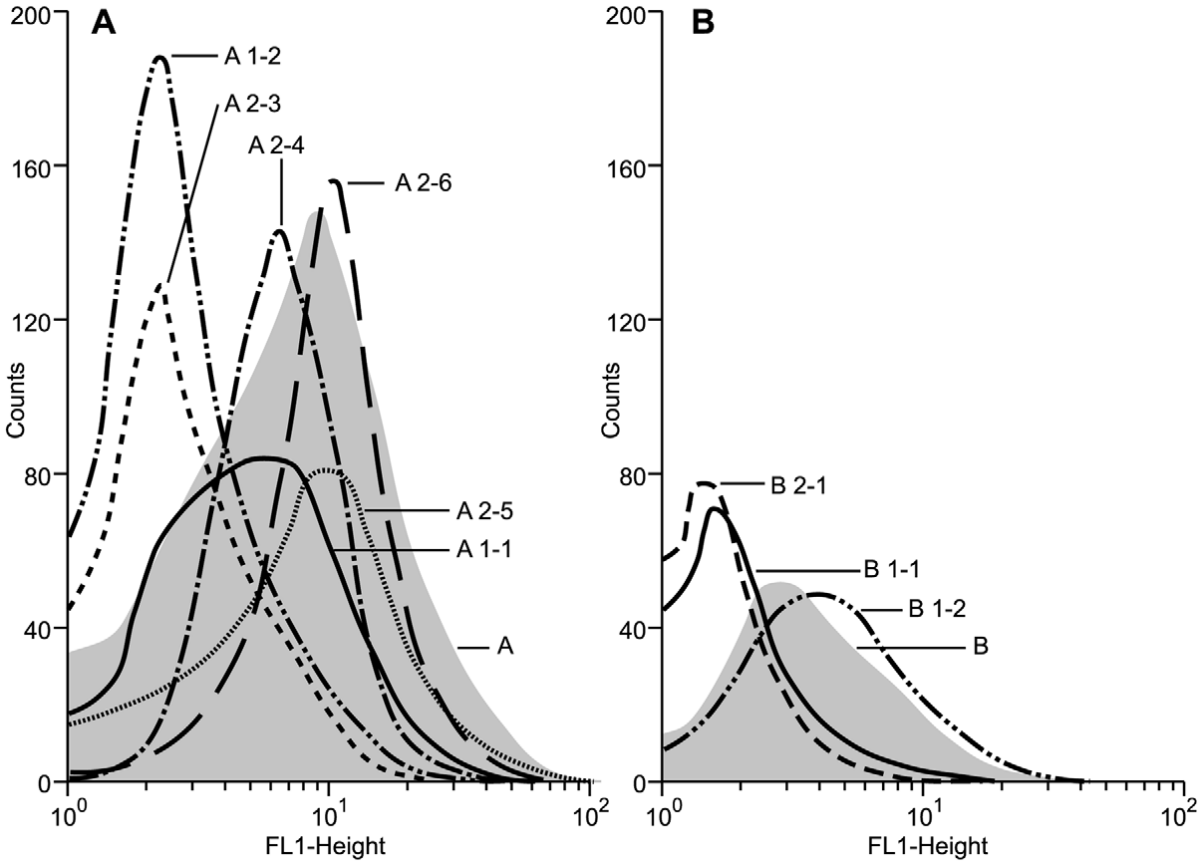
FACS analysis of EGFP expression in original and rederived cell lines. Green fluorescence generated by the transgenic bovine cells was determined using the FL1 emission channel. The relative numbers of viable cells was plotted as a function of variable intensities of green fluorescence from individual cells. (a) the original cell line A and all its rejuvenated derivatives and (b) cell line B and the cell lines that were rederived from it.

The original cell lines had distinct GFP expression profiles with a geometric mean fluorescence of 5.82 for cell line A and 3.30 for cell line B. The GFP expression profiles of the six rederived cell lines (A1-1, A1-2, A2-3, A2-4, A2-5 and A2-6) from fetuses A1 and A2 generated with donor cells from cell line A, were markedly different to the profile of the original cell line and varied considerably between the different cell lines (Fig. 2a). GFP expression levels ranged from a geometric mean fluorescence for cell lines A2-3 (6.08) and A2-5 (9.04) that were higher than the original cell line A (5.82), to geometric means of 2.47 for A1-2 and 2.36 for A2-6 which were similar to the fluorescence level measured for non-transgenic control cell lines and thus indicative of the absence of GFP fluorescence. A similar pattern of GFP expression profiles was observed for the original cell line B and its derivatives (Fig. 2b). Cell line B1-2 had a geometric mean fluorescence of 3.99, which was higher than the original cell line B (3.30) while the fluorescence levels of cell lines B1-1 and B2-1 were not above background levels (1.84 and 1.57, respectively).

### DNA methylation of transgene sequences in original and rederived cell lines

**Figure 3:**
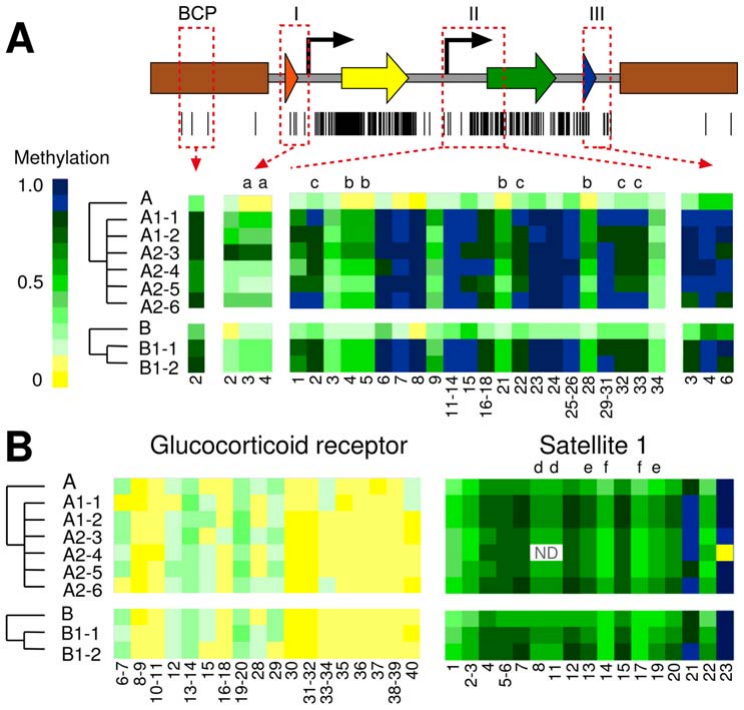
DNA methylation levels of transgene and endogenous genomic DNA sequences in original and rederived cells. (a) Schematic representation of the transgene construct (see Fig. 1 for a detailed description) depicting the location of the analyzed regions I, II, III and BCP. The black bars underneath indicate positions of CpGs within the transgene sequence. Methylation levels are shown as heatmap data with each square representing the average methylation level (expressed in proportional values with 0 = 0% methylated and 1 = 100% methylated) for a specific cleavage fragment. The numbers below the heatmap identify individual CpGs that are present on each of the analyzed cleavage fragments. The numbering itself refers to the order of the CpG sites as they appear in the DNA sequence. Alphabetic characters highlight fragments of the same mass/charge ratio which cannot be resolved by the QHTMS approach and only allows for conveying an average methylation value for these fragments. (b) Display of the methylation heatmap for two endogenous sequences in original and rederived cells. Numbers and alphabetical characters have been used as described under (a). ND: CpGs where the proportional methylation could not be determined.

The varied expression profile for the EGFP marker gene in the rederived cell lines, and the complete silencing of the puromycin selection marker in all of the rederived cell lines indicated that differences in the epigenetic modification of the transgene sequences may be present between original and rederived cell lines. DNA methylation profiles of the transgene sequences were therefore determined in all cell lines. Attempts were made to analyze the entire transgene construct, however, as is often the case in amplification of bisulfite converted DNA, in particular sequences of the highly GC-rich (73%) pac gene encoding the puromycin selection marker (not shown), this was not possible. The analysis therefore focused on four regions, representing important functional sections of the transgene construct (completely unmethylated prior to transfection). These regions included the loxP site and 5′ region of the first thymidine kinase (TK) promoter (region I), the second TK promoter and the 5′ region of the EGFP gene sequence (region II), the 3′region of SV40 polyA sequence and the lox2272 site (region III) and an endogenous sequence from the bovine β-casein promoter (BCP region) (Fig. 3a).

#### Region I–lox P and first TK promoter

Region I contained four CpG sites, two within the loxP site and two within the distal part of the TK promoter copy that drives the expression of the puromycin resistance marker gene. QHTMS analysis generated four CpG containing cleavage fragments, each containing a single CpG. One of the four cleavage fragments could not be analyzed due to a mass/charge ratio outside of the analyzable range and no information was therefore available for the 5′ most CpG of the loxP site. Average methylation across the three CpG sites analyzed revealed an almost complete absence of methylation across Region I in the original donor cell lines A and B (proportional methylation 0.05 and 0.06, respectively). The average methylation of these CpGs was consistently higher in all rederived cell lines compared to their original cell lines (Fig. 3a). Across all A-rederived populations the average of the proportional methylation was 0.36, indicative of hypermethylation of these CpGs in the rederived cell lines relative to the original cell lines (Table 1). Although slightly less pronounced, the same effect was observed for B-rederived cell lines with an overall proportional methylation average of 0.20.

#### Region II–second TK promoter and EGFP

The DNA sequence of region II contained a total of 34 methylation target sites, comprising of two CpGs within the intergenic linker sequence upstream of the TK promoter, 19 CpGs in the TK promoter sequence and the first 13 CpGs in the EGFP sequence. Of the 26 CpG containing cleavage fragments generated from Region II analysis, 22 fragments could be analyzed which provided information on 30 of the 34 CpGs. Some of the cleavage fragments contained multiple CpGs. In these cases the methylation proportion represents an average of the methylation level of all CpG sites present on a particular cleavage fragment. Overall, DNA methylation levels were low in original cell lines A and B with an average for the proportional methylation across all 30 analyzed CpGs of 0.09 and 0.17, respectively. This was in stark contrast with the methylation levels determined for the same DNA sequence in the rederived cell lines (Fig. 3a). Here, the average of the proportional methylation was 0.74 for the A-derived cell lines and 0.75 for the two B-derived cell lines B1-1 and B2-1 (Table 1). As was observed in Region I, the 22 fragments that were analyzed in Region II were hypermethylated in all rederived cell lines compared with the original cell lines. However, the increase in methylation is not equally distributed across all CpGs but rather is characterised by clusters of CpGs with very high methylation interspersed by CpGs that show only a moderate increase in methylation (Fig. 3a).

#### Region III-SV40 polyA and lox2272

Region III contained a total of six CpG sites, each in its own cleavage fragment. However, only three of the fragments had suitable mass/charge ratios which limited the measurement of the methylation levels to three CpG sites. Two of these informative CpGs were within a 27bp spacer region between the SV40 polyA sequence and the lox2272 site and the third one within the lox2272 sequence itself. Consistent with our results for regions I and II, the three CpGs in all rederived cell lines showed greatly increased methylation levels compared to the original cell lines A and B (Fig. 3a). While the original cell lines showed moderate methylation with an average of 0.36 and 0.47 for A and B, respectively, the average for the rederived cell lines was more than 0.9 (Table 1).

#### BCP region–β-casein promoter

While most parts of the transgene construct were comprised of sequences derived from organisms other than bovine, including bacteria and viruses, the β-casein promoter is of bovine origin and represents a sequence that is homologous to an endogenous gene locus. The assessment of methylation levels in this study was therefore limited to an average value for the two haploid copies of the endogenous gene locus and the additional copy(s) from the transgene. Compared to the different non-homologous sequence elements of the transgene, the β-casein promoter has a relatively low GC content (37%) and thus contains only a small number of CpGs. Analysis here was concentrated on the proximal promoter region. Unfortunately, the methylation profile of only one out of three CpG sites could be analyzed because the mass/charge ratio of the other two fragments that were generated was outside the analyzable range. Measurement of the level of methylation at this CpG site in the original cell lines A and B revealed a relatively low level of methylation for the β-casein sequence with 0.28 for A and 0.30 for B, respectively. Consistent with the observed hypermethylation in all rederived cell lines for regions I, II and III sequences, all rederived cell lines had elevated methylation levels of the CpG site within the bovine β-casein promoter. The average methylation was determined as 0.78 for derivatives of cell line A and 0.82 for B derived cell lines (Table 1). However, these values represent the average between the methylation at the endogenous locus and methylation of the transgene sequence. Although unlikely, the endogenous locus may contribute in part to the observed hypermethylation.

### DNA methylation analysis of endogenous loci in original and rederived cell lines

To determine whether the process of rederiving cell lines from a fetus following SCNT results in the general genome-wide hypermethylation of endogenous sequences, methylation levels of two endogenous sequences were measured.

#### Glucocorticoid Receptor (GR)

The GR-specific assay includes a region equivalent to the rat promoter exon 1_7_ that has been previously characterised to show differential methylation levels in response to a specific environmental stimulus in rats [9], [10], [11] and in humans [12]. Thus, it is a region that has the potential for dynamic changes of the levels of methylation. The endogenous GR locus that was examined in this study contains 40 CpG sites. Methylation analysis resulted in 20 CpG containing cleavage fragments with data able to be generated from 18 of these representing a total of 28 CpGs with some fragments containing single CpGs and others containing multiple CpGs on a single fragment (Fig. 3b).

In both original donor cell lines A and B, this region was almost unmethylated (proportional methylation 0.07 and 0.05, respectively). These methylation levels remained unchanged in all rederived cell lines compared with the original cell lines A and B (Fig. 3b, Table 1). The proportional methylation average measured was 0.06 for both the rejuvenated A-derived and B-derived cell lines.

#### Satellite 1 (Sat 1)

Sat 1 sequence is an abundant repeat element in the bovine genome and normally found in heterochromatin regions with a reasonably high degree of DNA methylation [13]. Although part of a heterochromatin domain, the sequence is not refractory to changes of its methylation level [13], [14].

The Sat 1 sequence analyzed contained a total of 23 CpGs. Of the 21 CpG-containing cleavage fragments that were generated, 17 fragments could be analyzed and provided information on the methylation status for 19 of the CpGs present in the Sat 1 sequence (Fig. 3b).

The Sat 1 repeat sequence was found, on average, to be moderately methylated in both original donor cell lines (A = 0.56 and B = 0.57). The proportional methylation for all of the rederived cell lines (average of 0.66 for A-rederived cells and 0.69 for B-rederived cells) were not significantly different (A-derived cell lines P = 0.09 and B-derived cell lines P = 0.42) to the original cell lines (Table 1). Analysis of three additional endogenous gene sequences (Fig. S1, Table S1) mirrored the results for the Sat 1 and GR sequences providing further evidence that endogenous sequences are not affected by differential methylation in original and rederived cells.

**Table 1:** Average methylation of the analyzed DNA sequences in rederived and original cell lines.

Cell line	DNA sequence					
	I	II	III	BCP	GR	Sat 1
A, original	0.05^c^	0.09^a^	0.36^a^	0.28^a^	0.07	0.56
A1-1	0.40	0.79	0.99	0.88	0.05	0.71
A1-2	0.40	0.75	0.99	0.90	0.06	0.70
A2-3	0.79	0.79	0.95	0.85	0.07	0.63
A2-4	0.14	0.71	0.97	0.63	0.04	0.60
A2-5	0.10	0.67	0.96	0.63	0.07	0.63
A2-6	0.33	0.76	0.88	0.82	0.05	0.70
Average rederived	0.36^d^	0.74^b^	0.97^b^	0.78^b^	0.06	0.66
B, original	0.06^e^	0.17^e^	0.47^e^	0.30^e^	0.05	0.57
B1-1	0.20	0.74	0.94	0.78	0.06	0.68
B2-1	0.19	0.75	0.92	0.86	0.05	0.71
Average rederived	0.20^f^	0.75^f^	0.93^f^	0.82^g^	0.06	0.69

ab values for the methylation within a specific region with these superscripts differ significantly; P<0.0005.

cd values for the methylation within a specific region with these superscripts differ significantly; P<0.026.

ef values for the methylation within a specific region with these superscripts differ significantly; P<0.009.

eg the difference of the methylation values with these superscripts are just outside the significance level (P<0.052). The P-value is 0.007 with a t-test assuming equal variances.

## Discussion

The introduction of complex modifications into livestock genomes has proven to be challenging because tools such as embryonic stem cell technologies are not available for efficiently performing targeted gene insertions in species other than mice [1]. One of the big hurdles in the alternative methodology, namely SCNT, is the limited lifespan of primary cell cultures [15], [16]. Complex site-specific modifications, such as cassette exchange, require a multi-step approach whereas the proliferative capacity of the primary cells is often already exhausted after a single selection step, restricting the option for the introduction of further modifications [17]. However, once selected, proliferative potential of the transgenic cells may be rejuvenated through SCNT and the rederivation of new, genetically identical, primary cells lines from cloned transgenic embryos [5], [18]. Rejuvenated cells would then be used for cassette exchange and ultimately for the generation of transgenic cows through SCNT. Although in theory such an approach appears ideal, for many applications success may depend on the faithful replication of selected parental cell lines including unaltered transgene functionality in the rederived cell lines.

The present study has investigated transgene expression in transgenic cell lines and their rejuvenated counterparts rederived following SCNT. However, despite being isolated from SCNT embryos generated from cells selected for their resistance to puromycin, the rederived transgenic cells were no longer resistant to this antibiotic. Further examination of transgene expression also identified that GFP expression levels varied considerably when compared to levels expressed by primary transgenic cells used for SCNT.

Silencing of transgenes is not a new observation [19], and has also been noted in SCNT rederived cells with silencing of neomycin and puromycin selection marker genes during a multi-step targeting approach in primary somatic cells [5]. However, although previous reports have often proposed that methylation of transgene sequences is the primary cause of such silencing, there is little documented evidence describing methylation status of silenced transgenes. Here we report on the methylation status of a transgene sequence displaying variable levels of expression. Although it was not possible to interrogate methylation levels at each CpG site of the transgene, and methylation levels measured at some CpG sites were reported as averages of multiple sites due to the nature of the technologies available, these limitations do not affect the overriding findings of the study. We observed some very limited methylation of the previously completely unmethylated transgene that had occurred following transfection and puromycin selection of cultured cells. In contrast to the limited transgene methylation that occurred during this initial in vitro culture phase, following the epigenetic reprogramming that must occur during SCNT and subsequent embryonic development, hypermethylation was seen across the transgene. Consistent with gene expression data, the occurrence of transgene hypermethylation was independent of transgene insertion site and was observed in two independent transgenic lines containing transgenes inserted into different random chromosomal locations. Moreover, the apparent hypermethylation of regions II and III was independent of the tissue from which transgenic cells were rederived following SCNT and was detected with similar degrees in a selection of cell lines of various embryonic and extra embryonic origins. For region I, cells derived from extra embryonic tissues (A2-4, A2-5, A2-6) appeared to have slightly lower methylation levels compared to cells derived from the embryo proper. However, as region I also showed by far the highest variability in the levels of methylation the observed difference may just indicate a DNA sequence subject to more promiscuous methylation.

The observed hypermethylation was not uniform across the entire length of the transgene, with some CpG sites showing notably greater increases in proportional methylation than others. Previous studies [20] have suggested that the primary sequence itself contributes to hypermethylation in transgenes. Although this may be true to some extent in this case, as much of the transgene is CpG rich, at least some of the hypermethylation measured in these studies was independent of the actual sequence. Evidence for this sequence independent methylation is provided by the two lox sites in the transgene, their sequences are virtually identical, however, they displayed varying degrees of hypermethylation. The 5′ loxP site showed only moderate hypermethylation (average of 32%) which was notably less than the 3′ lox2272 site which was on average 95% methylated in all rederived cell lines. Further evidence supporting the hypothesis that hypermethylation is not solely dependent on DNA sequence is the observation that hypermethylation appeared to extend into the 3′ region of BCP, which is a portion of the transgene consisting of homologous sequences derived from an endogenous bovine gene locus. The reason for hypermethylation occurring in the BCP transgene sequence is difficult to determine with great certainty, and analysis is complicated by the presence of two endogenous BCN copies in addition to the one or two transgenic copies present in the cells. However, consistent with much lower methylation levels prior to SCNT rederivation in the transgenic cell lines A and B (approximately 30%), analysis of only the endogenous BCP sequences in an equivalent non-transgenic cell line was found to have methylation levels far lower than the transgenic cell lines following SCNT (data not shown). This strongly suggests that selective hypermethylation of the BCN transgene sequence has occurred. Thus, while we have measured 80% as the average proportional methylation across all BCN sequences, the actual value for the transgene specific BCN sequences is likely to be closer to 100%. Moreover, the observed hypermethylation is not induced by a large array of transgene repeats, previously shown to have the potential of triggering transgene silencing and attracting methylation [21], [22], as the cell lines subject of this study only contain one or possibly two transgene copies.

It is believed that the hypermethylation observed across the transgene was specific to the transgene itself and not simply due to global reprogramming errors. Evidence supporting this hypothesis is the analysis of one endogenous single copy gene (GR) and one endogenous repeat sequence (Sat 1). Both GR and Sat 1 sequences displayed levels of methylation equivalent to levels previously measured in control samples [13] suggesting that the remainder of the genome had been appropriately reprogrammed and the process of SCNT itself was not simply causing genome wide hypermethylation.

The consequences of transgene hypermethylation and corresponding altered gene expression present a new hurdle in producing transgenic cattle. Rederived cells, originally selected for the presence of an antibiotic selection marker gene conferring resistance to the antibiotic puromycin became completely susceptible to the antibiotic, indicating reduced expression of the selection marker gene (driven by the pTK promoter) to levels below the threshold required for puromycin resistance. Notably, absence of resistance was observed even in cell lines A2-4 and A2-5 that showed only a relatively modest increase in the methylation of two CpG sites (3 and 4, region I) located within the 5′ section of the pTK promoter that drives expression of the puromycin resistance gene. This may indicate that hypermethylation of some or all of the 16 CpGs in the more 3′ part of the promoter are more critically important for the activity of this promoter. In contrast, although expression of the EGFP reporter gene was under control of an identical pTK promoter, expression of EGFP varied considerably compared to the original transgenic cell lines. Some rederived cell lines showed markedly lower EGFP levels while some displayed more expression, in spite of the fact that all rederived cells lines were rather uniformly hypermethylated in the pTK promoter (CpGs 3–18, region II) and 5′ region of the EGFP gene (CpGs 21-34, region II). These data indicate that hypermethylation of transgene sequences as a consequence of the SCNT process required for rederivation of cells and the generation of transgenic cows may not necessarily result in the absence of transgene expression and interfere for example with the intended production of a therapeutic protein – but does add an additional layer of complexity and uncertainty.

Unpredictable and often suppressed transgene expression, caused by hypermethylation and loss of antibiotic resistance, are not the only extra hurdles that must be overcome in transgenic animal production using a cassette exchange based approach. Methylation of loxP was associated with reduced Cre-lox recombination and thus, may adversely affect the cassette exchange reaction itself [23]. Preliminary efforts in our laboratory to demonstrate efficient Cre-lox replacement of the transgene with a “gene of interest” in primary cells have proven difficult (data not shown). However, due to the complexities of the system, a more detailed evaluation will be required to determine whether hypermethylation of CpG sites in the loxP sequences is a major factor in limiting successful cassette exchange. Nevertheless, it highlights the potential of hypermethylation and altered transgene expression in rederived cells to impair or even prevent the use of specific functions that may be crucial for a particular multi-step strategy.

## Materials and Methods

### Animal studies

All animal studies were undertaken in compliance with New Zealand laws and were approved by the Environmental Risk Management Authority, New Zealand (ERMA Approval GMD 02028) and the Ruakura Animal Ethics Committee (AE Applications 10724 and 11689).

### DNA constructs

A 7.2 kb SalI-NheI fragment and a 3.5 kb HindIII-SalI fragment comprising 5′ and 3′ regulatory regions of the CSN2 locus were excised from a 17.9 kb DNA fragment containing the entire bovine CSN2 gene [24]. The transgene was constructed by cloning these fragments upstream and downstream, respectively, of a cassette [8] containing a puromycin-resistance gene and an enhanced-GFP (EGFP) gene, each driven by an individual thymidine kinase (pTK) promoter and flanked by a pair of incompatible loxP sites (see Fig. 1a).

### Preparation, culture, and transfection of primary bovine fibroblasts

Cell line BFFL5 was obtained from a day 70 female fetus by disaggregation of lung tissue as previously described [24]. BFFL5 cells (10×10^6^, passage 6) were electroporated under 400 volts and 250 µF conditions with 10 µg of the gel-purified transgene fragment excised from the plasmid vector with Sal I. Transfected cells were diluted 1∶50 and passaged into 96-well plates. Puromycin selection (2 µg/ml) was applied 24 h post-transfection and maintained until cryopreservation.

While mock transfected cells did not result in any cell growth, cells transfected with the transgene fragment gave rise to 28 individual cell clones. Once the cell clones reached confluency they were trypsinized and passaged into a 24-well plate. Upon confluency, half the cells were cryopreserved and the remainder of the cells used for DNA extraction and PCR analyses.

### Analysis of individual cell clones

Presence of the transgene was confirmed in the isolated cell clones and all rederived cell populations by PCR and transgene copy number by quantitative real-time PCR as previously described [8]. Phenotypical EGFP expression of the cells was assessed by FACS analysis [8]. Resistance to puromycin of cultured cells was determined visually by observing the degree of cell death after 2 weeks of puromycin selection at 2 µg/ml. Cell clones A and B were selected as original cell lines for the rederivation study based on their low copy number (1–2 copies) and their proliferative capacity.

### Rederivation of cell lines following nuclear transfer

SCNT embryos derived from cell lines A and B were produced using a zona-free reconstruction method essentially as described in [25]. After in vitro culture for 7 days, either two or three SCNT blastocysts were transferred to synchronized recipients 7 days after estrus and pregnancy establishment determined at day 33 of gestation by trans-rectal ultrasound scanning. Pregnancies were terminated and fetuses recovered at day 35–48 of gestation for the rederivation of cell lines. Two fetuses (named A1 and A2) were obtained from recipients carrying SCNT embryos produced with cell line A. Following removal of head, tail and visceral organs, the embryos were cut into little tissue pieces which were ground through a stainless steel sieve (Sigma-Aldrich, St. Louis, MO) for further dissociation. For fetus A1, the resulting cell suspension and small pieces of tissue were cultured separately and gave rise to the rederived cell populations A1-1 and A1-2, respectively. After initially culturing equivalent cell populations for fetus A2, the two cell populations were later combined to generate cell line A2-3. In addition, cells contained in the allantoic and amniotic fluid of fetus A2 were collected and cultured to establish cell lines A2-4 and A2-5, respectively. Fetal cells were also grown out of small pieces of the amniotic sac which constitute the rederived cell population A2-6.

For cell line B, two SCNT embryos, designated B1 and B2, were recovered from one recipient. Both fetuses were processed as described above and dissociated cells and small tissue clumps were cultured together. The resulting rederived cell lines were designated as B1-1 and B2-1, respectively. In addition, cells from the amniotic fluid of fetus B1 were cultured to establish another cell line referred to as B1-2.

### DNA extraction

Total genomic DNA was extracted from all cell lines using Qiagen DNeasy extraction kit following the manufacture's protocol (Qiagen, Austin, TX). DNA concentration and purity was measured using the Nanodrop spectrophotometer (Thermo Scientific, DE, USA).

### Analysis of DNA methylation

QHTMS methodology [6], [7] was used to analyze DNA samples for the presence of methylated cytosines revealed by the methylation-dependent C to T transition following bisulfite treatment. The entire transgene sequence which was stably integrated into the genome of cell lines A and B was analyzed for the distribution of CpG dinucleotides as potential target sites for cytosine methylation using EpiDesigner (http://www.epidesigner.com/). Primers were designed for amplification of bisulphite-converted DNA sequences of CpG containing transgene regions as described [26]. Several endogenous loci with known methylation patterns [13], including the repeat sequence satellite 1 (Sat 1) and the glucocorticoid receptor gene sequence were used as control regions. The sequences of all primers used for the amplification of bisulphite-modified DNA are detailed in Table 2.

**Table 2:** PCR primer pairs used for DNA methylation analysis.

Fragment	Primer Sequence^a^	Amplicon size (bp)	Target sequence
I	TATTGGGTGGAAATATTTAGGTTTG	279	LoxP and start of TK promoter
	TACCTCAACCATAAAAACAAACACA		
II	GGATAGTAAGGGGGAGGATTG	520	Part of TK promoter and start of EGFP
	CAAATAAACTTCAAAATCAACTTACC		
III	GGGGTTTATTTAGGAAAATGAAATG	301	End of SV40 pA and lox2272
	TCCCCCTAAACCTAAAACATAAAATA		
BCP	TAAAAATTTTGGGGAGTATTTTAAAGG	461	β-casein promoter
	TAACAATCCACCAAACTCCACTATT		
GR	TTTTTTTGAAGTTTTTTTAGAGGG	324	Glucocorticoid receptor
	AATTTTCTCTATAATTTCTCTTCTTACC		
Sat 1	TGTAGATTGGGGATAGGAGAGTTAG	345	Satellite 1
	CCTACTTTATCTAAAAAAAATTACCTTCC		

a all forward primers (top) included the following common sequence at the 5′ end: AGGAAGAGAG; all reverse primers (bottom) the sequence CAGTAATACGACTCACTATAGGGAGAAGGCT.

DNA samples were analyzed using previously described methods [6], [7], [13]. Briefly, 1 µg DNA was bisulfite treated using the EZ-96 DNA Methylation gold kit (Zymo, CA, USA) to produce methylation-dependent sequence variations of C to T and regions of interest were amplified using T7 tagged PCR primers. PCR was performed using Platinum Taq DNA Polymerase (Invitrogen), with 1 µl bisulfite-converted DNA per reaction in a total volume of 10 µl. The PCR cycling conditions, recommended by Sequenom for amplification of bisulphite-treated DNA, were as follow: 94°C, 15 min followed by 45 cycles of 94°C, 20 sec; 56°C, 30 sec; 72°C, 1 min with a final extension at 72°C for 3 min. PCR products were analyzed by agarose gel electrophoresis to confirm successful amplification. In vitro amplification and transcription was performed on the reverse strand by T7 DNA and RNA polymerases and a simultaneous U specific cleavage by RNAse A. Approximately 20 nl of each sample was spotted onto Sequenom MassArray chips and subjected to mass spectrometry. The efficiency of bisulfite conversion was determined by assessing the quality of the raw data. This protocol enables precise and accurate high-resolution, high-throughput DNA methylation analysis, quantitative to 5% methylation for informative CpG dinucleotides [7]. A minimum of two independent DNA extractions, bisulphite conversions and PCRs were made for each of the amplicons analyzed, and as many as necessary to guarantee that at least two separate significant measurements of each CpG were recorded for statistical purposes.

Occasionally, incomplete bisulfite conversion was observed which can be readily identified as it produces additional, greater-than-expected peaks with mass/charge ratios of multiples of 16. Any data derived from incompletely converted DNA samples were excluded from the analyses.

The sequence-specific cleavage can produce fragments containing more than one methylated CpG site which the QHTMS approach is unable to resolve. In these cases, the measured methylation level does not represent the value for a single site but rather the average of the methylation level across all CpGs within that fragment. Similarly, processing of the samples can generate fragments with identical mass/charge ratios which are therefore indistinguishable and the methylation values will be averaged across the CpG sites contained on these fragments. Relevant information about fragments which were affected by averaging has been incorporated into Fig. 3 which summarizes the methylation results.

### Statistical analysis

The relative methylation of CpG sites were determined as previously described [13]. For each cell line, the average across all analyzed CpG sites for a specific region was calculated for each of the measurements done in duplicate. These averages were then used to calculate the average of the duplicate experiments. For the different cell lines rederived from cell line A and B, this average was subsequently used to calculate the average methylation value for all A-rederived and all B-rederived cell lines.

Pair-wise comparison of the average methylation values for original cell line versus rederived cell lines based on an unequal variance t-test was used to determine significance levels for the observed differences.

## Supporting Information

Figure S1 **DNA methylation levels for three endogenous genomic DNA sequences, Satellite II, Satellite alpha and SNRPN in original and rederived cells.** Methylation levels are shown as heatmap data with each square representing the average methylation level (expressed in proportional values with 0 = 0% methylated and 1 = 100% methylated) for a specific cleavage fragment. The numbers below the heatmap identify individual CpGs that are present on each of the analyzed cleavage fragments. The numbering itself refers to the order of the CpG sites as they appear in the DNA sequence. Alphabetic characters highlight fragments of the same mass/charge ratio which cannot be resolved by the QHTMS approach and only allows for conveying an average methylation value for these fragments. White squares depict CpGs where the proportional methylation could not be determined.
(TIF)

Table S1 PCR primer pairs used for the DNA methylation analysis of the endogenous Satellite II, Satellite alpha and SNRPN sequences.
(DOC)
