# Supplementary figures and images for: Primary Transgenic Bovine Cells and Their Rejuvenated Cloned Equivalents Show Transgene-Specific Epigenetic Differences
Source: PLoS One. 2012 Apr 20;7(4):e35619. doi: 10.1371/journal.pone.0035619 (PMC3332029; doi:10.1371/journal.pone.0035619)

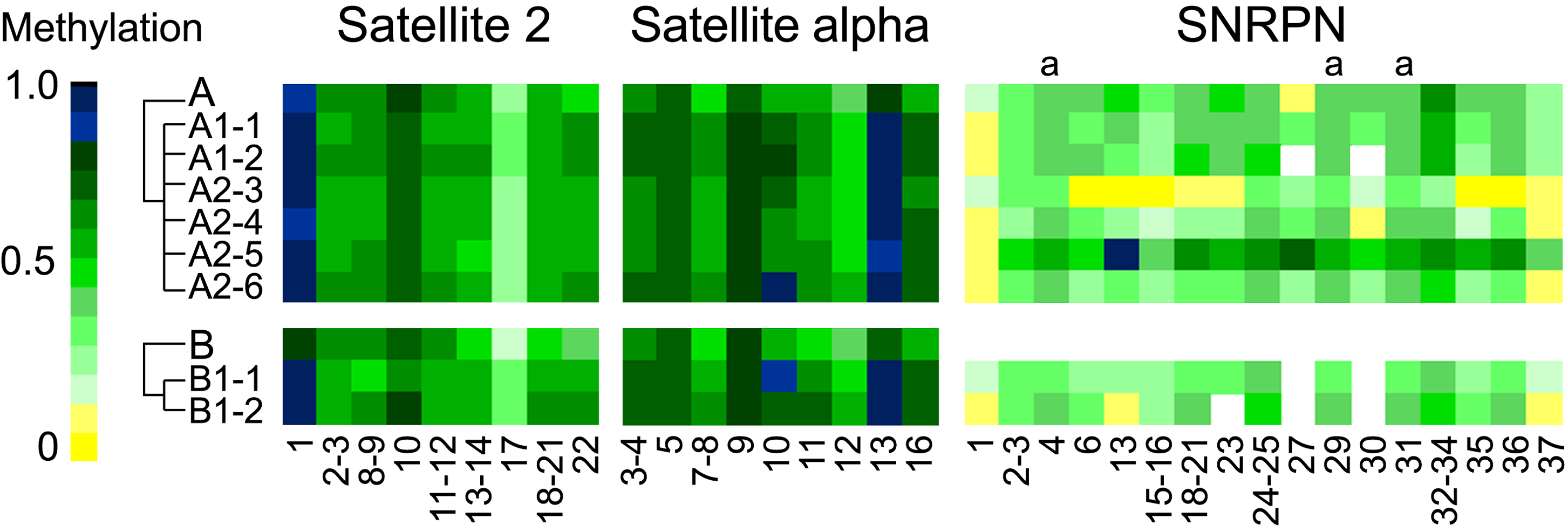

Supplement: Figure S1 — DNA methylation levels for three endogenous genomic DNA sequences, Satellite II, Satellite alpha and SNRPN in original and rederived cells. Methylation levels are shown as heatmap data with each square representing the average methylation level (expressed in proportional values with 0 = 0% methylated and 1 = 100% methylated) for a specific cleavage fragment. The numbers below the heatmap identify individual CpGs that are present on each of the analyzed cleavage fragments. The numbering itself refers to the order of the CpG sites as they appear in the DNA sequence. Alphabetic characters highlight fragments of the same mass/charge ratio which cannot be resolved by the QHTMS approach and only allows for conveying an average methylation value for these fragments. White squares depict CpGs where the proportional methylation could not be determined. (TIF) [file pone.0035619.s001.tif]
