# Supplementary material for: Primary Transgenic Bovine Cells and Their Rejuvenated Cloned Equivalents Show Transgene-Specific Epigenetic Differences
Source: PLoS One. 2012 Apr 20;7(4):e35619. doi: 10.1371/journal.pone.0035619 (PMC3332029; doi:10.1371/journal.pone.0035619)
Supplement: Table S1 — PCR primer pairs used for the DNA methylation analysis of the endogenous Satellite II, Satellite alpha and SNRPN sequences. (DOC) [file pone.0035619.s002.doc]

**Table S1.** PCR primer pairs used for the DNA methylation analysis of the endogenous Satellite II, Satellite alpha and SNRPN sequences.

| **Fragment** | **Primer Sequence^a^** | **Amplicon size (bp)** | **Target sequence** |
| --- | --- | --- | --- |
| Sat 2 | TTTGGTTTTAGGTTGGGAGTTTAAAG | 278 | Satellite 2 |
|  | AAAACAACAATCAAACACCACTCAC |  |  |
| Sat α | TTTTTTTTGATTTGGATAGGAGGG | 279 | Satellite alpha |
|  | ACTATATTTAAAACCAAAAATTTTTCC |  |  |
| SNRPN | TTGGGAGGTATTATTTTGGGTTGAA | 437 | SNRPN Gene |
|  | AACCCCAAACCTCCAAAAATTATC |  |  |

^a^ all forward primers (top) included the following common sequence at the 5’ end: AGGAAGAGAG; all reverse primers (bottom) the sequence CAGTAATACGACTCACTATAGGGAGAAGGCT
